# Retrograde Crossing Facilitating Rotational Atherectomy for Recanalization of Uncrossable Near Aorto-Ostial Chronic Total Occlusion: Case Report

**DOI:** 10.1016/j.jscai.2025.103942

**Published:** 2025-09-23

**Authors:** Min-Ping Huang, Chun-Ting Shih, Hsiu-Yu Fang, Shu-Kai Hsueh, Chiung-Jen Wu

**Affiliations:** Division of Cardiology, Department of Internal Medicine, Kaohsiung Chang Gung Memorial Hospital, Kaohsiung, Taiwan

**Keywords:** case report, chronic total occlusion, retrograde crossing, rotational atherectomy

## Abstract

Calcified near aorto-ostial chronic total occlusions pose considerable procedural challenges because of their intricate anatomical configuration. This case concerns a 64-year-old uremic woman with a near-ostial chronic total occlusion of the right coronary artery. Following the failure of retrograde RG3 externalization because of an uncrossable lesion, a staged percutaneous coronary intervention was undertaken, employing rotational atherectomy with a RotaWire Extra Support wire (Boston Scientific) that capitalized on microfractures previously created by antegrade and retrograde 0.014-inch guidewires. This case underscores the strategic utilization of guide wire–induced microfractures to facilitate direct RotaWire advancement and illustrates a safe and effective approach for navigating anatomically complex coronary lesions.

## Case presentation

A 64-year-old uremic woman presented with exertional chest tightness. Electrocardiography revealed symmetrical T-wave inversions in the anterior and inferior leads. Coronary angiography demonstrated a chronic total occlusion (CTO) near the ostium of the right coronary artery (RCA) and in-stent restenosis in the midsegment of the left anterior descending artery (LAD). Following percutaneous coronary intervention (PCI) with a drug-eluting balloon for the LAD lesion, the patient continued to experience chest discomfort during hemodialysis, prompting a subsequent PCI for RCA CTO recanalization.

**Figure 1:**
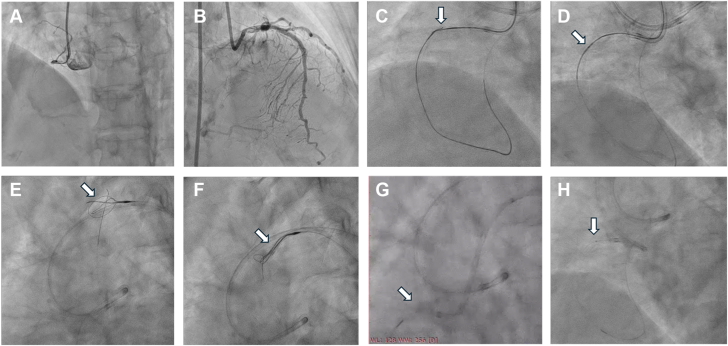
**Retrograde crossing and RG3 externalization.** (**A** and **B**) Coronary angiography demonstrated a chronic total occlusion at the ostium of the right coronary artery, with septal collaterals from the left anterior descending artery supplying the distal right coronary artery. (**C** and **D**) Alternating retrograde and antegrade punctures using stiff guide wires enabled retrograde wire passage into the antegrade guide catheter (GC); however, both the retrograde microcatheter and the smallest antegrade balloon failed to cross the lesion. (**E** and **F**) The retrograde RG3 guide wire successfully crossed the chronic total occlusion and was captured by a snare catheter within the antegrade GC. (**G** and **H**) Despite reengagement of the antegrade GC via the externalized RG3 wire and enhanced support, the smallest balloon still could not traverse the occlusion.

The left coronary artery was engaged with a 7F EBU 3.5 guide catheter (GC) (Medtronic), whereas the RCA was engaged using a 7F short-tip AL1 GC (Terumo Corp). Contralateral angiography demonstrated collateral flow from the septal branches of the LAD to the distal RCA and bifurcation of the posterior descending artery and posterolateral branch (Figure 1A, B).

A retrograde SUOH 03 guide wire (Asahi Intecc) supported by a Corsair Pro microcatheter (Asahi Intecc) was adeptly advanced through a septal branch to reach the distal CTO cap. Sequential wire escalation was performed using both antegrade (Gaia Next 1) and retrograde (Gaia Next 1, 2, and 4) approaches. Ultimately, a retrograde Conquest Pro 12 Sharpened Tip (CP12ST) (Asahi Intecc) successfully crossed the CTO and entered the antegrade guiding catheter (Figure 1C). However, neither the retrograde microcatheter nor a 1.0 × 5 mm balloon could traverse the severely calcified segment (Figure 1D).

A retrograde 0.010-inch RG3 wire was directly wired across the microfracture within the CTO and captured by an antegrade Atrieve Vascular Snare Kit (Argon Medical Devices) near the aortic arch for externalization (Figure 1E, F). Following reengagement of the antegrade GC over the RG3,^1^ despite multiple attempts, neither the Tornus catheter (Asahi Intecc) nor a 1.0 × 5 mm balloon could cross the lesion antegradely (Figure 1G, H). After 4 hours of intervention, because of operator and patient fatigue, the procedure was concluded.

**Figure 2:**
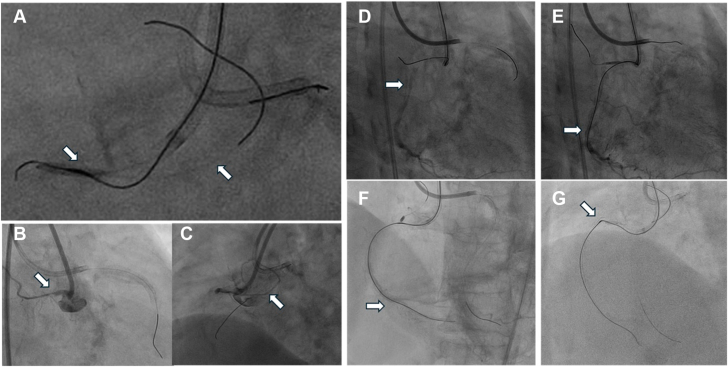
**Direct antegrade RotaWire wiring through the microfractures.** (**A**) A 2.0 × 15 mm balloon was partially inflated at the sinoatrial nodal artery near the right coronary artery ostium and partially within the guiding catheter to anchor the antegrade microcatheter. (**B**-**D**) The sinoatrial nodal artery was visualized in both right anterior oblique and left anterior oblique views, whereas contralateral angiography outlined the mid–right coronary artery. (**E**-**G**) The antegrade Conquest Pro 12 guide wire successfully crossed the chronic total occlusion, as confirmed by contralateral angiography; however, the microcatheter was unable to traverse the lesion.

During the second attempt, conducted 2 weeks later with the goal of performing rotational atherectomy, a 7F JR5 GC (Medtronic) was employed in place of the short-tip AL1 to achieve improved coaxial alignment with the RCA. After advancing a guide wire into the sinoatrial nodal artery, a 2.0 × 15 mm balloon was partially inflated at its origin near the RCA ostium and within the GC to anchor the antegrade Caravel microcatheter (Asahi Intecc), thereby supporting the advancement of a 0.014-inch XTA guide wire (Asahi Intecc) into the previously created microfractures (Figure 2A-D).

**Figure 3:**
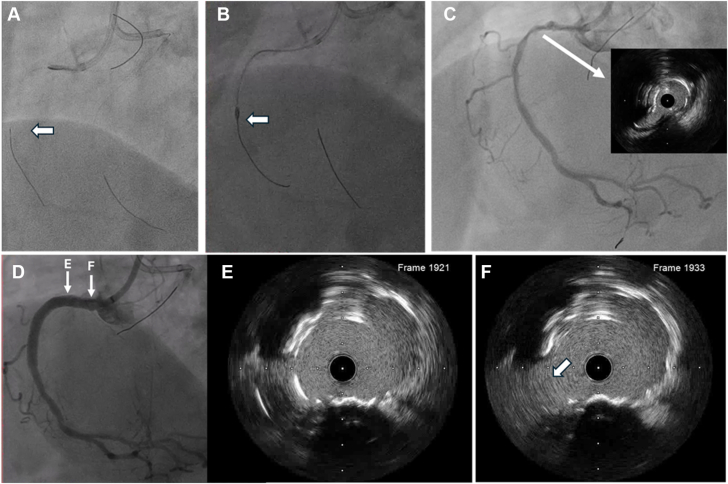
**Rotational artherectomy and RCA CTO recanalization.** (**A**-**C**) The antegrade RotaWire Extra Support guide wire successfully crossed the chronic total occlusion (CTO), enabling subsequent rotational atherectomy. Intravascular ultrasound confirmed a calcium fracture at the proximal CTO cap. (**D**-**F**) Following balloon angioplasty and a 3.5 × 48 mm stent deployment, the CTO was successfully recanalized. The arrow E highlights the stratified layer of the proximal CTO cap, while the arrow F indicates the plane of the sinoatrial nodal branch.

During antegrade guide wire escalation, a Conquest Pro 12 (CP12) successfully traversed the CTO via preexisting microfractures and entered the posterior descending artery, with true lumen entry confirmed by contralateral angiography (Figure 2E, F). As the Caravel microcatheter was unable to cross the lesion (Figure 2G), an antegrade RotaWire Extra Support guide wire (Boston Scientific) was advanced through the microfractures into the distal RCA, enabling rotational atherectomy with a 1.5 mm burr with speeds ranging from 170,000 to 148,000 rpm over 7 runs (Figure 3A-C).

Following serial predilatation with balloons and enhanced support via a 6F Guidezilla II guide extension catheter (Boston Scientific), a 3.5 × 48 mm drug-eluting stent was successfully deployed from the proximal to distal RCA, followed by postdilatation with a 4.0 × 15 mm high-pressure balloon. Final angiography confirmed successful recanalization of the CTO (Figure 3D-F).

## Discussion

Aorto-ostial CTO present considerable challenges because of proximal cap ambiguity and poorly defined vessel trajectories, often necessitating retrograde crossing strategies.^2^ Although coronary computed tomography angiography is recommended by the Asia-Pacific algorithm,^3^ the inherent instability of the antegrade system frequently impedes accurate balloon positioning either at the proximal cap or within the occlusion, limiting the feasibility of reverse controlled antegrade and retrograde tracking (reverse-CART) and antegrade dissection reentry techniques.^4^ Moreover, retrograde knuckled hydrophilic guide wire subintimal tracking poses an increased risk of iatrogenic aortocoronary dissection.^2^ Therefore, alternating antegrade and retrograde punctures using high tip-load guide wires to achieve an intraplaque rendezvous offers a more practical and controlled approach for aorto-ostial CTO.^4^ Unlike conventional CTO cases, where the retrograde wire primarily serves to identify the distal true lumen, its active role in lesion penetration is considerably enhanced in aorto-ostial CTO interventions. The microfractures created by both antegrade and retrograde guide wires enable subsequent direct wiring with smaller guide wires.

**Figure 4:**
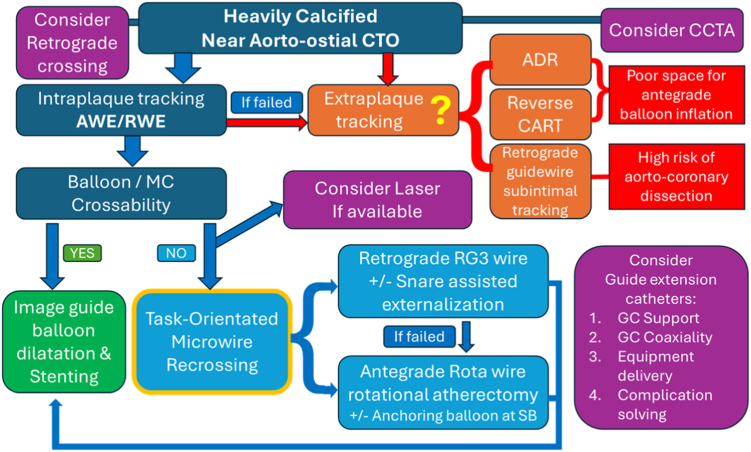
**The algorithm for heavily calcified near aorto-ostial chronic total occlusion (CTO) crossing.** Aorto-ostial CTO recanalization is challenged by complex anatomy and dense calcification, often precluding extraplaque strategies. A task-oriented direct wiring crossing the created microfractures offers a viable solution. This includes retrograde RG3 externalization with snare assistance for support, and antegrade RotaWire delivery for rotational atherectomy, often facilitated by side branch balloon anchoring. ADR, antegrade dissection reentry; AWE, antegrade wire escalation; CCTA, coronary computed tomography angiography; GC, guide catheter; MC, microcatheter; reverse CART, reverse controlled antegrade and retrograde tracking; RWE, retrograde wire escalation; SB, side branch.

**Figure 5:**
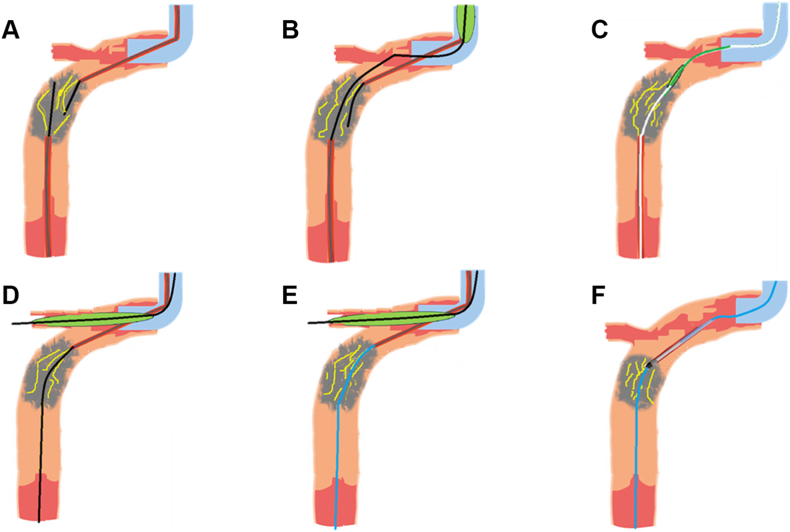
**Illustrated technique of rotational atherectomy enabled by guide wire–induced microfractures for near-ostial chronic total occlusion (CTO) crossing.** (**A**) A case of a near-ostial right coronary artery CTO, with multiple microfractures created by both antegrade and retrograde 0.014-inch guide wires (black). (**B**) The retrograde wire successfully traversed the heavily calcified CTO; however, the retrograde microcatheter was unable to cross. (**C**) The RG3 wire (white) achieved direct crossing through the previously created microfractures for externalization, yet even the smallest balloon failed to advance antegradely. (**D**) Following balloon anchoring at both the side branch and the antegrade microcatheter within the guide catheter, the antegrade guide wire successfully crossed the CTO via the preformed microfractures. (**E**) and (**F**) A RotaWire (blue) was advanced through the CTO along the preformed microfractures, enabling subsequent rotational atherectomy.

Direct wiring with retrograde 0.010-inch RG3 or antegrade 0.009-inch RotaWire Floppy/Extra Support guide wires serves several roles in PCI for uncrossable CTO. In one scenario, retrograde advancement of the RG3 guide wire through microfractures provides robust support via externalization.^5^^,^^6^ Alternatively, the antegrade RotaWire facilitates plaque modification through rotational atherectomy, enabling the subsequent delivery of intravascular imaging catheters and balloons^7^ as well as advanced lesion preparation techniques such as cutting or scoring balloons, high-pressure dilatation, and intravascular lithotripsy^4^ (Figures 4 and 5).

The snare technique, commonly used in retrograde CTO PCI, enables the capture of the retrograde RG3 guide wire within the antegrade guiding catheter. However, procedural challenges may arise during the release of the snared wire inside the guiding catheter and the withdrawal of the bent wire from the retrograde microcatheter, posing potential procedural risks.^5^

In this case, the direct wiring of the RotaWire Extra Support wire through the heavily calcified segment was pivotal to procedural success.^7^ Laser atherectomy eliminates the need to exchange the 0.014-inch guide wire for dedicated atherectomy wires; however, its availability remains limited across many centers.^8^ The Carlino technique, although proposed to facilitate guide wire and microcatheter advancement, is associated with lower success rates and more complications.^9^ The anchor balloon technique, deployed in the ostial side branch alongside the microcatheter, enhanced the stability of the antegrade guiding catheter for direct wiring.^4^

## Conclusion

Calcified near aorto-ostial CTO are marked by reduced success rates because of their complex anatomical configuration and often need retrograde crossing. Although both retrograde and antegrade microcatheters failed to traverse the lesion, the microfractures created by bidirectional 0.014-inch guide wires served as a strategic investment, permitting direct advancement of RotaWires. This technique has demonstrated the safety and feasibility in enabling subsequent rotational atherectomy and successful recanalization of these challenging lesions.
